# Model of the Austenite Decomposition during Cooling of the Medium Carbon Steel Using LSTM Recurrent Neural Network

**DOI:** 10.3390/ma14164492

**Published:** 2021-08-10

**Authors:** Adam Kulawik, Joanna Wróbel, Alexey Mikhailovich Ikonnikov

**Affiliations:** 1 Department of Computer Science, Czestochowa University of Technology, Dabrowskiego 73, 42-201 Czestochowa, Poland; joanna.wrobel@pcz.pl; 2 Department of Mechanical Engineering Technology, Polzunov Altai State Technical University, 46 Lenina Avenue, Barnaul 656038, Russia; iamagtu@mail.ru

**Keywords:** cooling, phase transformation in solid state, CCT diagrams, artificial neural network, LSTM network

## Abstract

The motivation of the presented paper is the desire to create a universal tool to analyse the process of austenite decomposition during the cooling process of various steel grades. The presented analysis concerns the application of Recurrent Artificial Neural Networks (RANN) of the Long Short-Term Memory (LSTM) type for the analysis of the transition path of the cooling curve. This type of network was selected due to its ability to predict events in time sequences. The proposed generalisation allows for the determination of the austenite transformation during the continuous cooling process for various cooling curves. As training data for the neural network, values determined from the macroscopic model based on the analysis of Continuous Cooling Transformation (CCT) diagrams were used. All relations and analyses used to build training/testing or validation sets are presented in the paper. The modelling with the use of LSTM network gives the possibility to determine the incremental changes of phase transformation (in a given time step) with the assumed changes of temperature resulting from the considered cooling rate.

## 1. Introduction

The basic property of Recurrent Neural Networks (RNN) is their ability to collect data and its subsequent processing. Recurrent networks differ from unidirectional networks by the presence of feedback loops, i.e., a loop connecting the output of a given neuron to its input. RNN is able to map target vectors from the entire history of the previous input data. A detailed description of a recurrent neural network can be found in Sherstinsky [1]. Unfortunately, one of the obstacles to using recurrent networks has been the problem of vanishing and exploding gradients.

The most popular and best-known variety of recurrent networks is the Long Short-Term Memory (LSTM) model, which is capable of training long-term dependencies and avoids the problem of vanishing gradients [2,3]. Many theoretical and experimental papers have been published on the application of LSTM networks in various scientific fields [4]. LSTMs are effective in capturing long-term temporal dependencies and are mainly used in language modelling, speech recognition, handwriting recognition and machine translation, or the analysis of audio and video data [5,6]. Thus, LSTM is able to consider data of different lengths and capture long-term relationships between them. It also finds applications in mechanics and metal heat treatment.

Li et al. [7] proposed a classification method based on discrete wavelet transform and LSTM network for finding and identifying faults type in mobile robot motor bearings. The presented model allowed for accurate identification of a fault type for different motor speeds. On the other hand, Zhao et al. [8] in their work presented a novel machine health monitoring system by combining Convolutional Neural Networks (CNN) with bi-directional LSTM networks. In the presented model, the CNN was designed to extract local reliable features, while bi-directional LSTM, which were built on the CNN, encoded the temporal information and trained the representations. It was shown that the proposed model does not require expert knowledge, and experimental results confirmed the excellent performance of the method for tool wear prediction. A bi-directional LSTM was also used by Zhang et al. [9]. The developed model was designed to eliminate noise interference and detect rail cracks. The obtained results showed that the presented model is effective at detecting crack signals in real applications. Liu et al. [10] applied the CNN-LSTM algorithm to detect defects in the molten pool. The CNN–LSTM algorithm extracted the basic features of the molten pool image and identified welding defects. The conducted experimental study showed that the built algorithm is universal and can be applied to similar image recognition and classification tasks. Conversely, Fernández et al. [11] presented in their work an ANN–LSTM architecture for the detection and classification of defects in the welding process based on video sequences. Additionally, Sudheera et al. [12] used LSTM to interpret ultrasonic signals to characterise welding defects. The large variation in the length of the processed input sequences affected the accuracy of the data. Jaypuria et al. [13] compared the performance of a recurrent neural network (RNN) and a back-propagation neural network (BPNN) for modelling the electron beam welding of AISI 304 stainless steel. Based on the calculations, they found that RNN, compared to BPNN, showed better prediction accuracy but lower computational speed. Gorji et al. [14] used a recurrent network with the GRU type to model the plane stress plasticity for arbitrary loading paths.

Taking the above characteristics of recurrent neural networks into account, they were selected to build an incremental model for determining phase transformations during the cooling process of steel elements. It was assumed that the knowledge base for the network would be the model of phase transformations.

The properties of steel resulting from its structural composition have a significant impact on the common use of steel by industry. To determine the type of steel structure, for example, iron-carbon systems are used [15]. In contrast, iron-graphite systems are rarely used in practice to determine the structure of steel because very slow cooling with high carbon content in the alloy is required for graphite nucleation. These technological conditions impose the high activation energy required for graphite formation. The metastable system is used to evaluate the microstructure of materials with carbon content between 0.0% (pure iron) and 6.6% (cementite). However, phase equilibrium systems are used to determine phase transformation products only at low supercooling. Theoretically, at a very low cooling rate, the steel transformations occur according to the iron-cementite diagram. In practice, higher rates, at which the supercooling phenomenon occurs, are used much more frequently. Increasing the cooling rate leads to a decrease in the transformation temperature and, consequently, to the joining of the lines $A_{r1}$ and $A_{r3}$. Increasing the heating rate leads to a higher transformation temperature and a larger difference between the lines $A_{c1}$ and $A_{c3}$. Since the phase equilibrium diagram is not suitable for estimating the material structure during rapid processes, the Time-Temperature Transformation (TTT) and Continuous Cooling Transformation (CCT) diagrams are used to determine the transformations when the material is supercooled. However, in heating processes (overheating), the Continuous Heating Transformation (CHT) diagram is used. These diagrams are built on the basis of dilatometric, magnetic, electric or acoustic tests. Based on these diagrams, it is possible to determine the start time of a given transformation, the end time of transformations under isothermal cooling (TTT diagram) and continuous cooling (CCT diagram), as well as the percentage share of individual phases. Even on the basis of experimental research, it is difficult to clearly determine the boundaries between the individual phases, as in reality, they are not clear. In CCT diagrams, these boundaries are usually given as conventional.

**Figure 1:**
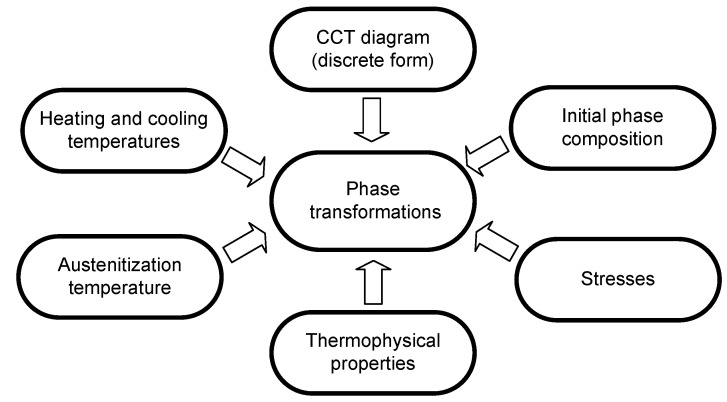
Basic elements affecting the kinetics of phase transformations in the solid state.

In modelling phase transformations in the solid state, a number of factors affecting the quality of the model must be taken into account (Figure 1).

An empirical macroscopic model based on the analysis of CCT diagrams and the Avrami and Koistinen–Marburger equations were chosen.

## 2. Model of Phase Transformations in Solid State—Building Sets for Neural Networks

The method of calculating the phase transformations in a solid state for the thermal treatment processes may use data from the process of the isothermal or continuous cooling (TTT or CCT diagram) and heating (CHT diagram). These diagrams contain information on the decomposition of austenite during continuous or isothermal cooling, showing the temperature-time ranges of the transformation of supercooled austenite. As already noted, these curves are obtained using microstructural, dilatometric, acoustic and other tests [16,17]. Temperature-time curves during heating or cooling are approximated by a sequence of temperature-time steps. In each step, the contribution of the new phase is calculated from the kinetics of the transformation, which is modelled according to the laws of Johnson–Mehl–Avrami (JMA) [18].

Volumetric fractions of phases $\eta_{\left(\cdot \right)}\left(T,t\right)$ formed during cooling are estimated from Avrami’s formulas, taking into account the share of the austenite formed in the heating process

(1) $$\begin{array}{c} \eta_{\left(i\right)}\left(T,t\right)\;=\;min\left\{\;\eta_{i}^{\%},{\tilde{\eta }}_{A}-\sum_{j\neq i}\eta_{j}\right\}\left(1\;-\;exp\left(-b\left(T\right)t^{n(T)}\right)\right)\\ \mathrm{or}\\ \eta_{\left(i\right)}\left(T,t\right)\;=\;\eta_{i}^{\%}\cdot \left({\tilde{\eta }}_{A}\;-\;\sum_{j\neq i}\eta_{j}\right)\left(1\;-\;exp\left(-b\left(T\right)t^{n(T)}\right)\right) \end{array}$$

where $\eta_{i}$ is the share of the *i*-th phase created in the cooling process, and $\eta_{i}^{\%}$ is the final share of the phase (*i*) estimated from the CCT diagram.

The values of the coefficients n(T) and b(T) are estimated from the solution system of two equations for start $\left(\eta_{s},t_{s}\left(T\right)\right)$ and end of the transformations $\left(\eta_{f},t_{f}\left(T\right)\right)$ [19,20].

(2) $$\begin{array}{cc} n\left(T\right)\;=\;\frac{ln\left(\frac{ln(1-\eta_{f})}{ln(1-\eta_{s})}\right)}{ln\left(\frac{t_{f}\left(T\right)}{t_{s}\left(T\right)}\right)}, & b\left(T\right)\;=\;\frac{-ln(1-\eta_{s})}{{\left(t_{s}\right)}^{n(T)}} \end{array}$$

The share of the formed martensite is determined on the basis of the empirical Koistinen–Marburger Equation [21]

(3) $$\eta_{M}\left(T,t\right)\;=\;\left({\tilde{\eta }}_{A}\;-\;\sum_{i\neq M}\eta_{i}\right)\left(1\;-\;exp\left(-k{\left(M_{s}-T\right)}^{n}\right)\right)$$

where $M_{s}$ is the temperature of the martensitic transformation start, k≅0.015, n = 1 (for medium carbon constructional steel) [21].

The value of the *k*-factor is calculated on the basis of the formula

(4) $$k=-\frac{ln(1-\eta_{MAX})}{M_{s}-M_{f}}$$

where $\eta_{MAX}\;=\;0.99$ (assumption) is the maximum share of martensite, $M_{s}$ is the starting temperature, and $M_{f}$ is the finish temperature of martensitic transformation.

The Koistinen–Marburger equation provides a good approximation of the kinetics of martensite formation after a full austenitic transformation.

Taking into account the kinetics of phase transformations described by Equations (1)–(3), the share of the austenite transformation temperature during the cooling process is defined by the relation

(5) $$\eta_{A}\left(t,T,\eta_{F},\eta_{P},\eta_{B},\eta_{M}\right)\;=\;1\;-\;\left(\eta_{F}\left(t,T,\eta_{A}\right)\;+\;\eta_{P}\left(t,T,\eta_{A}\right)\;+\;\eta_{B}\left(t,T,\eta_{A}\right)\;+\;\eta_{M}\left(t,T,\eta_{A}\right)\right).$$

**Figure 2:**
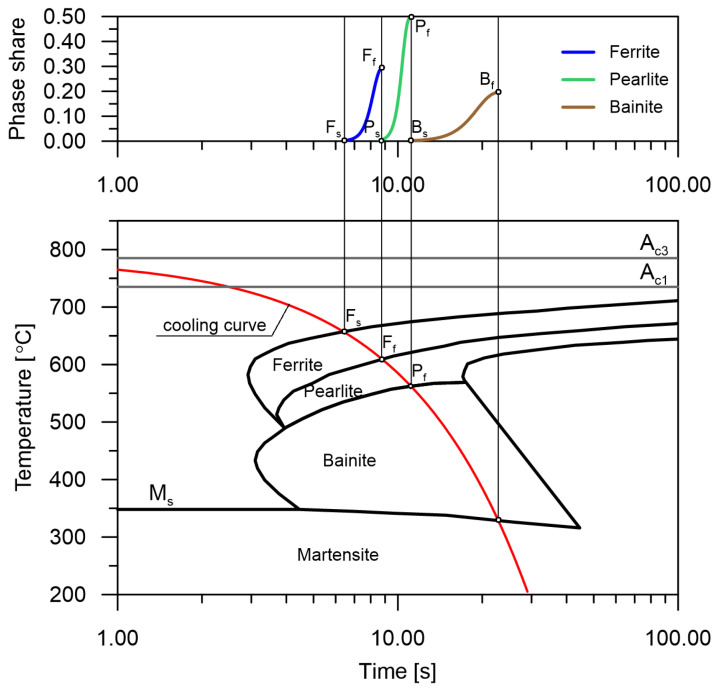
The determination of ranges of phase transformations from the CCT diagram.

When analysing the CCT diagrams, it can be assumed that the lines indicate the start or the finish of phase transformations (Figure 2). However, it should be noted that these points can mean more than just boundaries between particular transformations but also places where the kinetics of the entire transformation can change.

**Figure 3:**
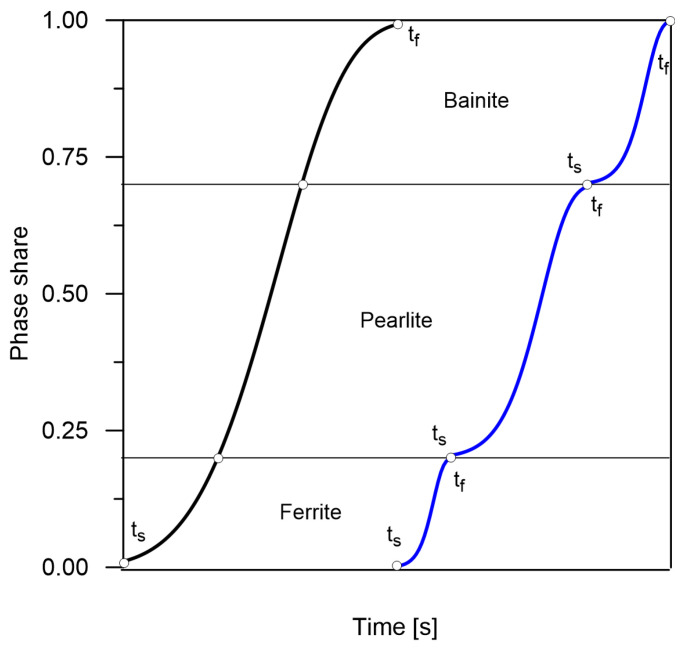
Difference in kinetics of phase transformations—a model of joint and separated transformations.

One of the approaches (from the point of view of the CCT analysis) is to assume that the phase transformations proceed sequentially according to their own, unrelated kinetics (the model of kinetics of separate transformations—the *3S* model). In a different approach to the analysis of time-temperature-transformation diagrams, it is assumed that individual phase transformations (only diffusion transformations) follow one global kinetics model (the *1S* model) (Figure 3) [19].

Several assumptions should be made for the global kinetic model. If the phase transformation during cooling is the first (based on the time determined from the intersection of the temperature curve with the CCT diagram), then (based on CCT analysis) the transformation start time and transformation end time as well as the maximum percentage of transformation are determined. Based on these data, the end time is calculated for the individual phase transformations.

(6) $$t_{f}\left(t,\eta^{\%},t_{s}\right)\;=\;exp\left(\frac{-A\times (ln\left(t_{s}\right)-ln\left(t\right))}{B(\eta^{\%})}\right)\;\times \;t_{s}$$

If the phase transition during cooling is the last on the basis of the time of the designated intersection of the temperature curve with the CCT diagram, determine the transformation start time, then estimate the transformation end time. On the basis of these data and the current level of the share of phases occurring before the considered transformation, the start time of all transformations necessary for the formula for the shares of phases is determined.

(7) $$t_{s}\left(t,\eta^{\%},t_{f}\right)\;=\;{\left(\frac{t^{\frac{A}{B(\eta^{\%})}}}{t_{f}}\right)}^{\left(\frac{B(\eta^{\%})}{A-B(\eta^{\%})}\right)}$$

If the phase tranformation occurs between two others, then based on its start time, estimated end time, estimated maximum share of the phase and the level of the preceding phase, the start and end time of global transformation is determined

(8) $$\begin{array}{c} t_{s}\left(t_{1},\eta_{1}^{\%},t_{2},\eta_{2}^{\%}\right)\;=\;{\left(\frac{{\left(t_{2}^{\frac{A}{B\left(\eta_{2}^{\%}\right)}}\right)}^{N_{2}}}{{exp\left(\frac{A}{B\left(\eta_{1}^{\%}\right)}\cdot ln\left(t_{1}\right)\right)}^{N_{2}}}\right)}^{N_{1}} \end{array}$$

where

(9) $$N_{1}\left(\eta_{1}^{\%},\eta_{2}^{\%}\right)\;=\;\frac{1}{\left(-\frac{A}{B\left(\eta_{1}^{\%}\right)}+1\right)*\frac{B\left(\eta_{2}^{\%}\right)}{A-B\left(\eta_{2}^{\%}\right)}+1}$$

(10) $$N_{2}\left(\eta_{2}^{\%}\right)\;=\;\frac{B\left(\eta_{2}^{\%}\right)}{A-B\left(\eta_{2}^{\%}\right)}$$

(11) $$A\;=\;ln\left(\frac{ln(1-\eta_{f})}{ln(1-\eta_{s})}\right)$$

(12) $$B\left(\eta^{\%}\right)\;=\;ln\left(\frac{ln(1-\eta^{\%})}{ln(1-\eta_{s})}\right).$$

**Figure 4:**
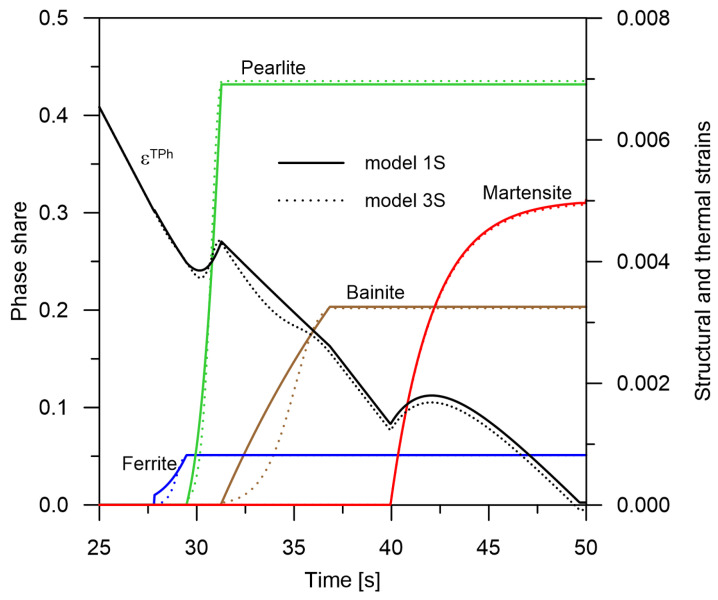
A comparison of the kinetics of transformations and the strains $\varepsilon^{T}$ + $\varepsilon^{ph}$ for *1S* and *3S* models.

In order to show the differences in the kinetics of the phases obtained from the proposed models, the shares of individual phases for the cooling rate 30 °C/s were determined (Figure 4).

The CCT diagrams used in the model were made in such a way that the time of the onset of austenite decomposition is 0 when the cooling curve reaches the $A_{c3}$ temperature. The real time of the intersection of the $A_{c3}$ line by the cooling curve is denoted as $t_{s}^{0}$. This assumption means that the course (time, rate) of the cooling curve above the temperature $A_{c3}$ is not significant and that it is possible to directly compare the diagrams for different austenitising temperatures. Since the diagrams were made in laboratory conditions for specific constant cooling rates, it is possible to analyse CCT diagrams in several ways to determine the start and end times of transformations.

**Figure 5:**
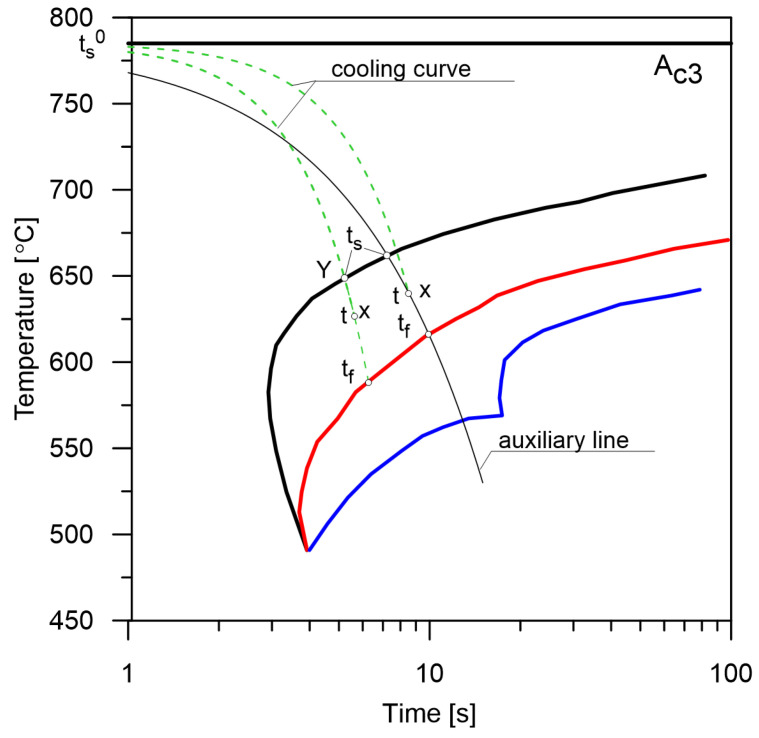
CCT diagram analysis when cooling at different rates.

**Method No. 1** (the average cooling rate from $A_{c3}$, Figure 5).

It is assumed that the share of a particular phase can be estimated by drawing a cooling line for a constant rate passing through the end point of the cooling curve. The next steps of this method are as follows:

- determine the time (point X) by subtracting the intersection time $A_{c3}$ ($t_{s}^{0}$) from the current time of the phase transformation;
- carrying out an auxiliary line connecting point X with point ($t_{s}^{0}$,$A_{c3}$);
- determine the intersection points between the line and the curves of the start and end of the transformation and determine the $t_{s}$ and $t_{f}$ times.

**Method No. 2** (the average cooling rate starting from the actual start time of the transformation, Figure 5).

The share of the phase transformation is calculated on the basis of the actual time of intersection of the cooling curve and the start line of the transformation. The next steps of this method are as follows:

- determine the start time of the transformation $t_{s}$ (point Y);
- determine the time t (point X);
- carrying out an auxiliary line connecting point X with point Y;
- determine the intersection point between the line and the curve determine the end of the transformation and $t_{f}$.

**Method No. 3** (the double-pass method).

The next steps of this method are as follows:

- determine the temperature curve over the entire range of cooling;
- determining the intersection points between the cooling curve and the lines of the CCT diagram;
- determine the kinetics of phase transformations.

The double-pass method has one fundamental disadvantage: taking into account the phase-temperature coupling is very difficult and inefficient. After every phase increase, which leads to a temperature change, an update of the cooling curve is required.

In the presented paper, method No. 2 was used to analyse the cooling curve. The presented model for computing phase transformations in the solid state based on the analysis of CCT diagrams as well as the Avrami and Koistinen–Marburger equations was the starting point for a model giving the same results and implemented in an artificial neural network. Hypothesis: The use of RNNs to determine phase transformations during continuous cooling will allow for greater universality of the model. There will be no problems resulting from classical calculations, for example, regarding the separation line of transformations crossing several times or changes of cooling rates in the areas of phase transformations.

As input data to the recurrent neural network model—the training, testing and validation sets—have been determined on the basis of the models presented above. The data for each set were determined for constant cooling rates, that is, for identical conditions to those for which the CCT diagram was performed.

## 3. Details of Experimental Procedure

It was assumed that a recurrent neural network would approximate the phase transformations (austenite decomposition curves) at an appropriate level without the need for complex models. Because the austenite decomposition curve is a function of time and temperature changes, it cannot be approximated using traditional neural networks. It is assumed that each next time step is a change in temperature level by some certain value. In the analysed model, the training data are the next cooling steps, where, for a constant value of the cooling rate, we have a constant change of the temperature value. This approach allows taking into account the cooling history. The input data of recurrent neural networks are an array of the temperature values as a function of time, while the output data are an array of austenite concentrations. This approach allows for changes in the cooling rate at each time step of the calculation. It is assumed that the time step is constant. However, there is no objection to consider the values of austenite concentrations for smaller time steps proportionally to the size of the time step.

On the basis of the curves representing austenite decomposition during cooling, as obtained from the analysis of CCT diagrams, different architectures of recurrent networks were analysed (Table 1). The first analysed element is the number of LSTM layers. It was assumed that each layer is connected to the next one by all outputs. In order to calculate the cost function for all results in time steps, a TimeDistributed layer was used as the last one. In addition, the effect of adding a layer of Dense type as an intermediate layer between the LSTM layer and the TimeDistributed layer was analysed. The influence of the batch size was also analysed, assuming the sizes of 30, 100, 128 and 300, respectively. As expected, a small batch size of results had increased accuracy at the expense of computation time. On the other hand, a large batch size significantly accelerated the calculation, often increasing the flattening area on the training curve, sometimes to several hundred epochs. This resulted in the necessity to significantly increase the number of epochs in order to obtain the same accuracy as the neural network. In this paper, it was decided to analyse only one batch size. It was determined that the number of input sequences inserted into the neural network during one iteration (taking into account the accuracy and time of analysis) will be 100. Thus, the number of iterations per one epoch was equal to 18. For a smaller number of data per iteration, it did not significantly increase the accuracy for a given number of epochs.

**Figure 6:**
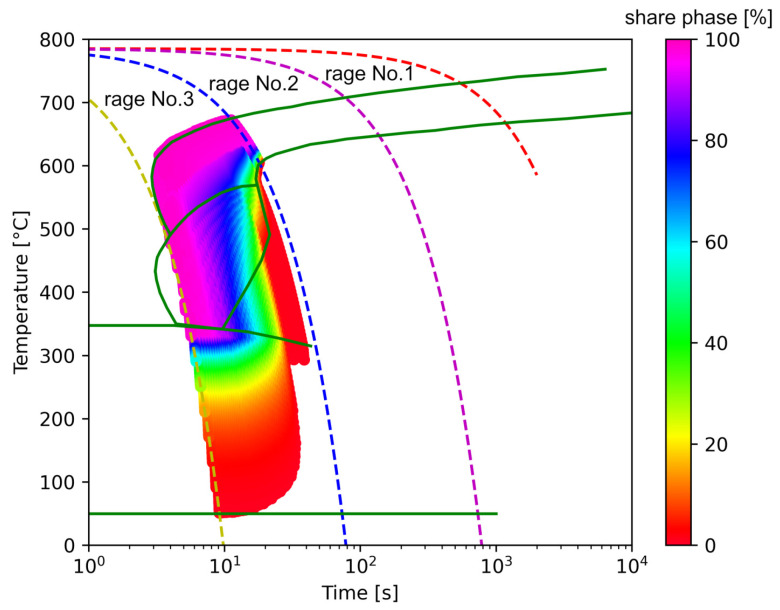
CTP diagram divided into analysed ranges—input/output data for RNN.

The areas of transformation analysis were divided into three ranges according to the rate scheme: range No. 1 is the area of transformations with average cooling rates from 0.1 to 1 K/s, range No. 2 from 1 to 10 K/s and range No. 3 from 10 to 80 K/s (Figure 6). It was assumed that for the range with the highest rates, the temperature value during the cooling process should decrease by 800 K, while for the first two ranges, only by 200 K. This division allowed taking into account the total transformation of austenite into phase transformations during the cooling process for C45 medium carbon steel. The first and second ranges were the phase transformations of austenite → ferrite and pearlite and the third range austenite → ferrite, pearlite, bainite and martensite. For each of the areas, it was assumed that the time step would be selected so that the number of data in the time series did not exceed 200 steps for one cooling rate and was not less than 20 (Table 2).

Based on the tests of the model, it was determined that, as expected, the greatest accuracy problem would be in the third range. Therefore, only results for the area with the most complicated austenite decomposition function were presented in this paper.

**Figure 7:**
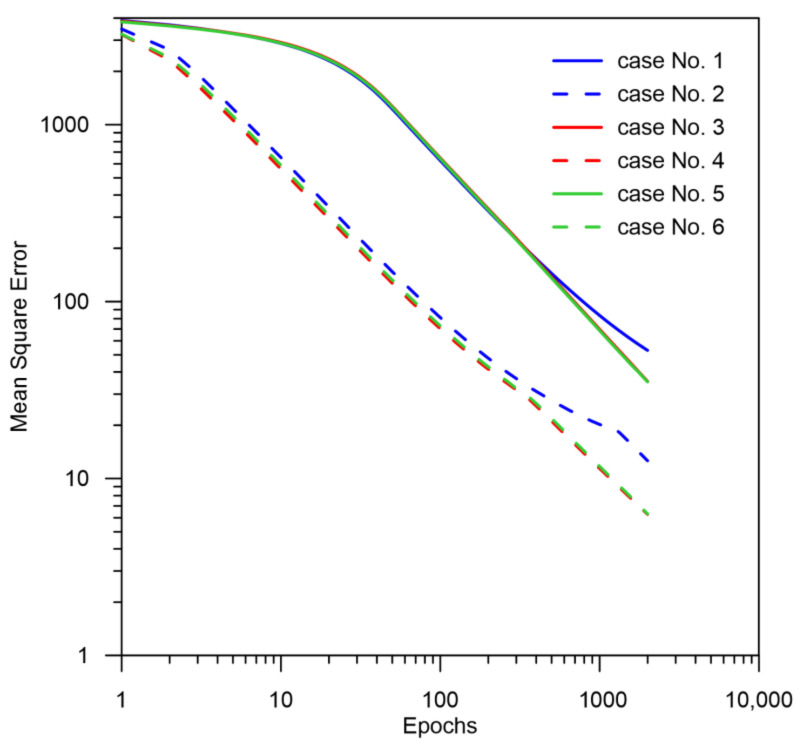
The training error history for different network architectures.

The aim of this paper is to approximate the austenite decomposition curve during the cooling process with the following assumptions:

- the analysis starts when the $A_{c3}$ temperature exceeds 1058 °C (t = 0 s);
- the number of epochs 2000 allowed to analyse the training process to the point, in which the training level does not improve and there is no error reflection on the validation data indicating overfitting (Figure 7);
- the number of input data was equal to 3600 files;
- the set was divided proportionally into a 50% training set, 25% a testing set and 25% a validation set. Data were assigned to each set randomly without repetition.

## 4. Examples of Calculation

**Figure 8:**
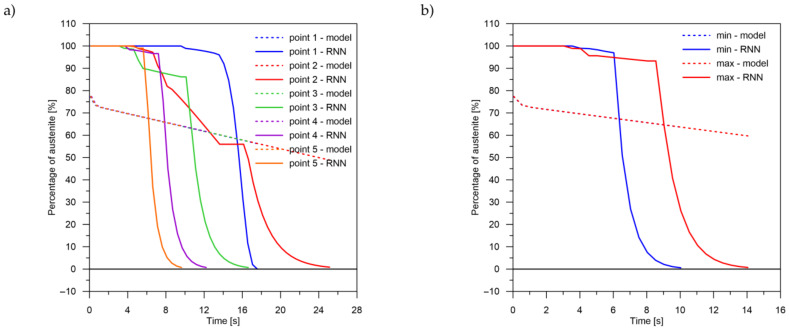
Kinetics of transformations: a comparison of the analytical model with the RNN model for case No. 1. (**a**) Control rates. (**b**) Rates with the largest (max—positive, min—negative) errors.

**Figure 9:**
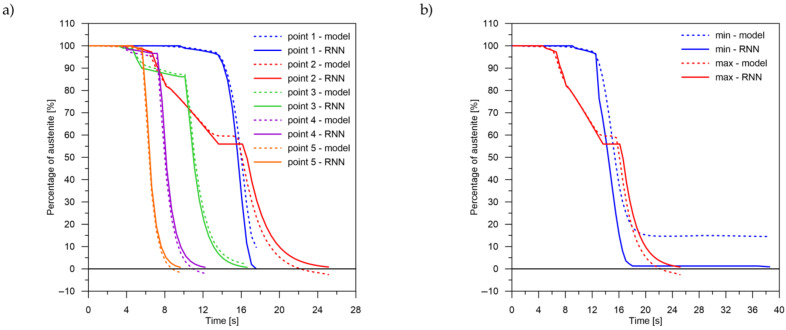
Kinetics of transformations: a comparison of the analytical model with the RNN model for case No. 2. (**a**) Control rates. (**b**) Rates with the largest (max—positive, min—negative) errors.

**Figure 10:**
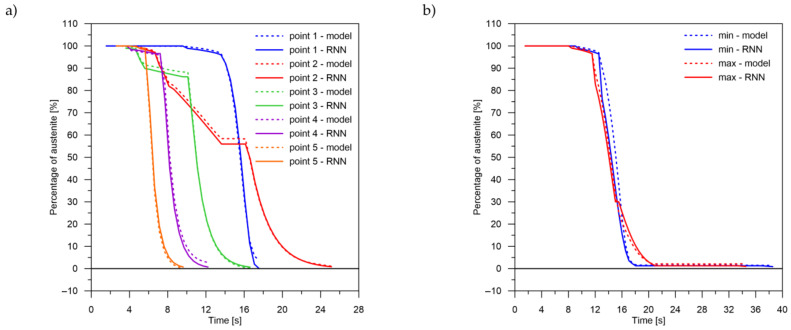
Kinetics of transformations: a comparison of the analytical model with the RNN model for case No. 3. (**a**) Control rates. (**b**) Rates with the largest (max—positive, min—negative) errors.

**Figure 11:**
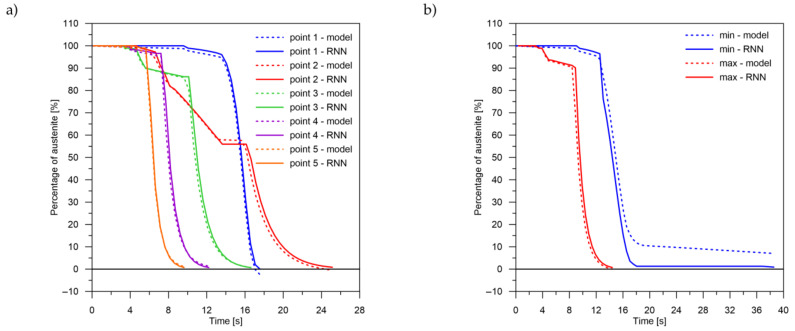
Kinetics of transformations: a comparison of the analytical model with the RNN model for case No. 4. (**a**) Control rates. (**b**) Rates with the largest (max—positive, min—negative) errors.

**Figure 12:**
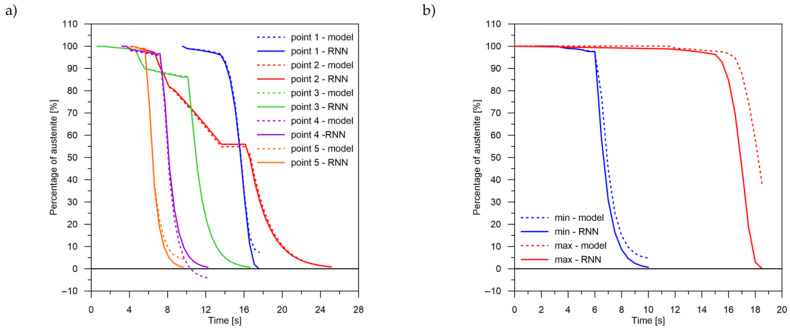
Kinetics of transformations: a comparison of the analytical model with the RNN model for case No. 5. (**a**) Control rates. (**b**) Rates with the largest (max—positive, min—negative) errors.

**Figure 13:**
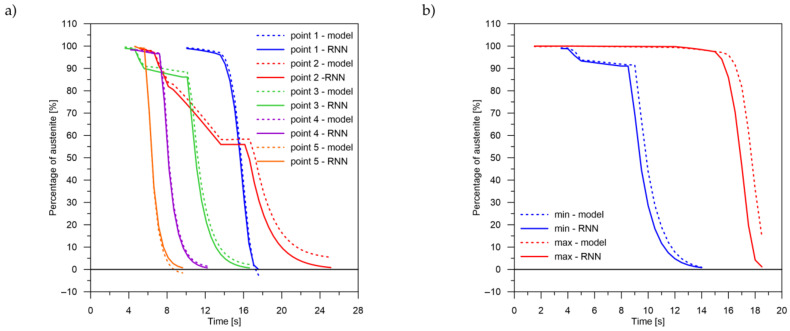
Kinetics of transformations: a comparison of the analytical model with the RNN model for case No. 6. (**a**) Control rates. (**b**) Rates with the largest (max—positive, min—negative) errors.

The values of the austenite transformation were determined at control points for each case of the considered network geometry and presented in the Figure 8, Figure 9, Figure 10, Figure 11, Figure 12 and Figure 13. As control points, the kinetics of austenite transformation were determined for a given constant cooling rate for range No. 3 (point 1—cooling rate 11.5 K/s, point 2—cooling rate 28.3 K/s, point 3—cooling rate 45.0 K/s, point 4—cooling rate 61.7 K/s and point 5—cooling rate 78.4 K/s). In addition to the comparison of the considered cases at the control points (Figure 8a, Figure 9a, Figure 10a, Figure 11a, Figure 12a and Figure 13a), the kinetic curves for the greatest undershoot and the greatest overshoot (Figure 8b, Figure 9b, Figure 10b, Figure 11b, Figure 12b and Figure 13b) were also presented. The largest undershoot was defined as the largest difference in the transformations determined by the recursive network in relation to the analytical model, resulting in a decrease in the transformation value over time. This difference was calculated locally for a given time step.

The main feature of convolutional networks or densely connected networks is the lack of a memory mechanism. The incremental processing characteristic of the human brain cannot be modelled by such networks. This type of processing is possible by recurrent neural networks. However, the idea of processing differs significantly from the classical incremental model with computing previous historical data. The time sequence of data is treated by recurrent networks as a single observation. It uses a mechanism of processing a new element of the data sequence by restoring the initial value of the state based on an inner loop. Due to the problem of gradient fading, assuming that the time sequences will have between 81 and 139 analysed elements, LSTM recurrent layers were chosen. To examine the ability of recurrent networks to create representations, cases consisting of several layers—from 2 to 5 layers—were analysed. All sublayers returned a complete sequence of output objects. The output layer was a TimeDistributed layer, which is a wrapper of a dense layer with unit output. This means that a complete merge operation is performed on each time step.

The ADAM efficient optimization algorithm and mean square error loss function were used [22]. The rectified linear unit activation function (ReLU) was used at the output of the network. All calculations were performed using the Keras open-source software library [23,24].

## 5. Results and Conclusions

At the beginning, it should be noted that during the analysis of time series, the quality of the obtained results can be determined in at least several methods. The first one may concern the analysis of the entire changes in time and obtaining the final value (in this case, the level of a given phase) at the end of the process (after cooling). However, this type of analysis does not give us many important conclusions. The second type of analysis can only concern the reaction of the described model based on RNN at a given time step. This means how large the differences are between the respective time steps (local increase/decrease in the contribution of the phase transformation). These local changes and differences may or may not finally lead to a difference in the levels of the particular transformations after the process; they may just balance each other. The third analysis may be concerned with differences in kinetics over time or, more precisely, with the rate at which a particular level of phase transformation is reached. In this case, the differences are not in the level of transformation but in the time to achieve a given level of transformation. This type of information is fundamental in determining, for example, the level of internal stresses that depend on changes in time. Looking through the prism of these three methods of analysis, several conclusions can be reached.

The obtained results—both the final levels of transformations and their changes in time—for a smaller number of LSTM layers (2 layers) indicate that a recurrent network with this geometry is not able to approximate the curves of the austenite decomposition (Figure 8). The difference can not only be seen in the kinetics of the transformations but also in the difference between the histories of the error generation in the training process (between two and more layers) (Figure 7, Figure 8, Figure 9, Figure 10, Figure 11, Figure 12 and Figure 13).

**Figure 14:**
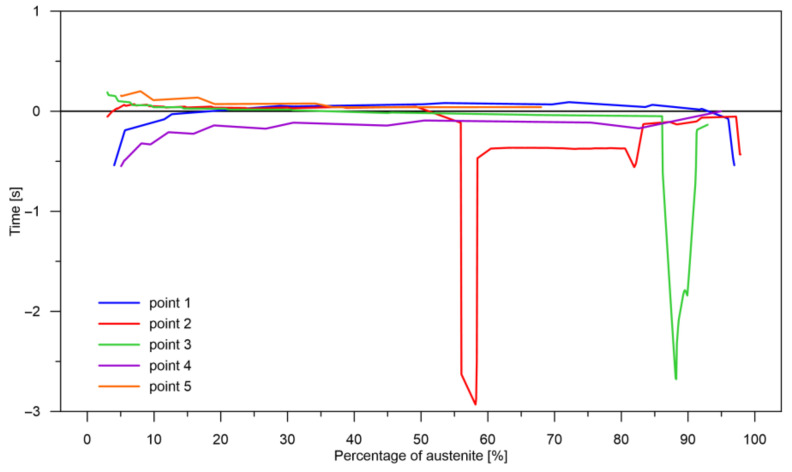
Time to achieve the required level of transformation for case No. 3 (difference between analytical and RNN model—control rates).

**Figure 15:**
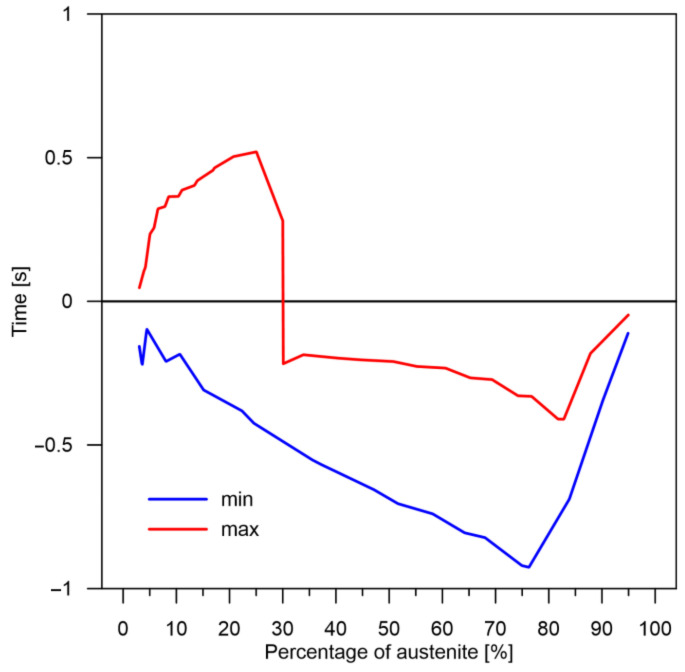
Time to achieve the required level of transformation for case No. 3 (difference between analytical and RNN model—rates with largest errors).

The decomposition curves, especially for the area where decomposition into ferrite, pearlite, bainite and martensite occurs, are strongly non-linear. It is not a challenge to model the phase transformations in the area of carbon diffusion (range No. 1 and range No. 2) (Figure 6). The reduction of the error value is only achieved by adding a Dense layer (Figure 9) to two LSTM layers. The network behaves much better after adding additional LSTM layers. Adding a Dense layer decreases the error value calculated during network training. However, this does not contribute to the accuracy of the obtained results in such a way that this solution can be recommended. As can be observed from the error results, especially calculated as differences in local transformation changes in time steps, the best approximation is obtained for three LSTM network layers without the additional layer (Figure 10). The use of more layers causes strong oversizing of the network. Furthermore, as a result, the additional layer leads to an increase in the error values for both undershooting and overshooting the estimated values. In modelling time series, the instantaneous error value (Table 3) is often important but also the time for which the austenite level value is matched to the correct (Figure 14 and Figure 15). Such timing inaccuracies can cause small as well as large strain variations. They can lead to both small and large error values of critical parameters, for example, temporary stress levels. It is expected that such small differences between transformation levels should not result in an increase in stresses, but this requires further investigation. A local analysis focusing on a single time-space node will not give an answer in the case of stress analysis. In this case, the difference in kinetics between the different nodes will be important and will magnify the stresses. It also seems that the trained network should respond well to varying cooling lines. The paper focused on modelling the process of phase transformations in the solid state when assuming the change in temperature level (cooling rate) is constant (3600 time invariant different cooling lines). Such generalisation by a neural network seems obvious, but this also requires further work. It should also be noted that it is surprising that there is no large difference in the MSE error in the training process for such qualitatively different results obtained in the paper (Figure 7, Figure 8, Figure 9, Figure 10, Figure 11, Figure 12 and Figure 13). During the training process, the authors more often analysed the matches used for identification than the presented MSE error. It seems that this type of measure even when analysing the change in real numbers can give interesting results.

In conclusion, the obtained results for the method of austenite distribution analysis using recurrent LSTM layers confirm that the applied methodology makes sense. It also allows replacing in complex mathematical models where the essence is the processing of incremental data occurring in time a universal way.

## Figures and Tables

**Table 1:** Selected architectures of the analysed recurrent neural networks.

Layer	Number of Layer					
	Case No. 1	Case No. 2	Case No. 3	Case No. 4	Case No. 5	Case No. 6
LSTM (100, 83, 83)	2	2	3	3	5	5
Dense (100, 83, 83)	0	1	0	1	0	1
TimeDistributed (100, 83, 1)	1	1	1	1	1	1

**Table 2:** Time distribution for the different areas of rate analysis.

Cooling Rate (K/s)	ΔT (K)	Time (s)	Time Step (s)	Number of Time Steps to Complete the Calculation
0.1	200	2000	10	200
1	200	200	10	20
1	200	200	1	200
10	200	20	1	20
10	800	80	0.5	160
80	800	10	0.5	20

**Table 3:** Residual standard deviation.

Case No.	Point 1	Point 2	Point 3	Point 4	Point 5	Max	Min
1	34.52	30.53	36.59	40.43	42.06	38.86	42.34
2	5.58	3.70	2.24	3.22	2.10	4.78	12.62
3	1.71	1.36	1.28	2.91	1.49	2.13	4.92
4	1.92	2.08	2.60	3.04	1.27	5.31	7.11
5	2.90	0.96	0.20	3.50	2.85	26.17	7.10
6	2.71	5.21	3.63	1.72	1.95	16.28	8.41

## Data Availability

The data presented in this study are available at https://icis.pcz.pl/~jwrobel/MDPI_data.zip (accessed on 8 August 2021).

## References

[1] Sherstinsky A. (2020). Fundamentals of Recurrent Neural Network (RNN) and Long Short-Term Memory (LSTM) network. Phys. D Nonlinear Phenom..

[2] Hochreiter S., Schmidhuber J. (1997). Long short-term memory. Neural Comput..

[3] Gers F.A., Schmidhuber J., Cummins F. (2000). Learning to forget: Continual prediction with LSTM. Neural Comput..

[4] Van Houdt G., Mosquera C., Nápoles G. (2020). A review on the long short-term memory model. Artif. Intell. Rev..

[5] Greff K., Srivastava R.K., Koutník J., Steunebrink B.R., Schmidhuber J. (2017). LSTM: A search space odyssey. IEEE Trans. Neural Netw. Learn. Syst..

[6] Zaremba W., Sutskever I., Vinyals O. (2014). Recurrent neural network regularization. arXiv.

[7] Li S., Zhao Y., Ding M. (2018). Mobile robot motor bearing fault detection and classification on discrete wavelet transform and LSTM network. J. Mech. Med. Biol..

[8] Zhao R., Yan R., Wang J., Mao K. (2017). Learning to Monitor Machine Health with Convolutional Bi-Directional LSTM Networks. Sensors.

[9] Zhang X., Zou Z., Wang K., Hao Q., Wang Y., Shen Y., Hu H. (2018). A new rail crack detection method using LSTM network for actual application based on AE technology. Appl. Acoust..

[10] Liu T., Bao J., Wang J., Zhang Y. (2018). A Hybrid CNN–LSTM Algorithm for Online Defect Recognition of CO_2_ Welding. Sensors.

[11] Fernández A., Souto A., González C., Méndez-Rial R. Embedded vision system for monitoring arc welding with thermal imaging and deep learning. Proceedings of the 2020 International Conference on Omni-layer Intelligent Systems (COINS).

[12] Sudheera K., Nandhitha N.M., Sai V.B., Kumar N.V. (2020). Deep Learning Techniques for Flaw Characterization in Weld Pieces from Ultrasonic Signals. Russ. J. Nondestruct. Test.

[13] Jaypuria S., Gupta S.K., Pratihar D.K., Shunmugam M., Kanthababu M. (2020). Comparative Study of Feed-Forward and Recurrent Neural Networks in Modeling of Electron Beam Welding. Advances in Additive Manufacturing and Joining.

[14] Gorji M.B., Mozaffar M., Heidenreich J.N., Cao J., Mohr D. (2020). On the potential of recurrent neural networks for modeling path dependent plasticity. J. Mech. Phys. Solids.

[15] Blicharski M. (2012). Inżynieria Materiałowa.

[16] Dobrzański L. (2007). Podstawy Kształtowania Struktury i Własności Materiałów Metalowych.

[17] van Bohemen S.M.C., Sietsma J., Hermans M.J.M., Richardson I.M. (2003). Kinetics of the martensitic transformation in low-alloy steel studied by means of acoustic emission. Acta Mater..

[18] Avrami M. (1939). Kinetics of phase change. I General theory. J. Chem. Phys..

[19] Kulawik A. (2013). Modelowanie Zjawisk Obróbki Cieplnej Stali średniowęglowych.

[20] Pan J., Gu J., Gur C., Pan J. (2009). Mathematical Fundamentals of Thermal Process Modeling of Steels. Handbook of Thermal Process Modeling Steels.

[21] Koistinen D.P., Marburger R.E. (1959). A general equation prescribing the extent of the autenite-martensite transformation in pure iron-carbon alloys and plain carbon steels. Acta Metall..

[22] Kingma D.P., Ba J.L. EADAM: A method for stochastic optimization. Proceedings of the 3rd International Conference on Learning Representations (ICLR 2015).

[23] Chollet F. (2017). Deep Learning with Python.

[24] Keras—Simple. Flexible. Powerful. https://keras.io/.

